# Mass Spectrometric Identification of *In Vivo* Phosphorylation Sites of Differentially Expressed Proteins in Elongating Cotton Fiber Cells

**DOI:** 10.1371/journal.pone.0058758

**Published:** 2013-03-13

**Authors:** Bing Zhang, Jin-Yuan Liu

**Affiliations:** Laboratory of Molecular Biology and MOE Key Laboratory of Bioinformatics, School of Life Sciences, Tsinghua University, Beijing, P. R. China; Lawrence Berkeley National Laboratory, United States of America

## Abstract

Two-dimensional gel electrophoresis (2-DE)-based proteomics approach was applied to extensively explore the molecular basis of plant development and environmental adaptation. These proteomics analyses revealed thousands of differentially expressed proteins (DEPs) closely related to different biological processes. However, little attention has been paid to how peptide mass fingerprinting (PMF) data generated by the approach can be directly utilized for the determination of protein phosphorylation. Here, we used the software tool FindMod to predict the peptides that might carry the phosphorylation modification by examining their PMF data for mass differences between the empirical and theoretical peptides and then identified phosphorylation sites using MALDI TOF/TOF according to predicted peptide data from these DEP spots in the 2-D gels. As a result, a total of 48 phosphorylation sites of 40 DEPs were successfully identified among 235 known DEPs previously revealed in the 2-D gels of elongating cotton fiber cells. The 40 phosphorylated DEPs, including important enzymes such as enolase, transketolase and UDP-L-rhamnose synthase, are presumed to participate in the functional regulation of numerous metabolic pathways, suggesting the reverse phosphorylation of these proteins might play important roles in elongating cotton fibers. The results also indicated that some different isoforms of the identical DEP revealed in our 2-DE-based proteomics analysis could be annotated by phosphorylation events. Taken together, as the first report of large-scale identification of phosphorylation sites in elongating cotton fiber cells, our study provides not only an excellent example of directly identifying phosphorylation sites from known DEPs on 2-D gels but also provides a valuable resource for future functional studies of phosphorylated proteins in this field.

## Introduction

Protein phosphorylation is one of the most widespread regulatory mechanisms in biological systems. Reversible phosphorylation governs protein conformation and functions, thereby affecting nearly all cellular activities including signal transduction, gene expression, cell cycle progression and other biological processes [1], [2], [3]. Its importance can be judged by the fact that approximately 30% of all eukaryotic proteins are estimated to undergo phosphorylation modification [4]. Among the amino acids that can be phosphorylated, *O*-phosphates are the most abundant and mostly attached to serine, threonine and tyrosine residues with a pSer/pThr/pTyr ratio in the order of 1800∶200∶1 [3], [4]. Furthermore, phosphorylation is often a sub-stoichiometric process. Thus, typically at any given time, not all copies of a given protein/site are in the phosphorylated state, resulting in a dynamic profile during biological processes [4], [5]. Therefore, the identification and quantitative determination of *in vivo* phosphorylated proteins/sites is of outstanding importance for understanding biological processes.

Due to their important biological significance, the identification of phosphorylated proteins/sites is increasingly becoming the central point of proteomics research. During the last decade, the use of mass spectrometry combined with phosphopeptide enrichment techniques, such as immobilized metal affinity chromatography, titanium dioxide column chromatography and strong cation exchange chromatography, for the global characterization of phosphorylation modification has become the most common approach for studying phosphorylated peptides/sites in higher organisms [5], [6]. Various large-scale phosphoproteomics studies have been carried out in yeast [7], mice [8], humans [9], plants [6] and other species [10] using this gel-free approach. On the other hand, two-dimensional gel electrophoresis (2-DE)-based comparative proteomics approach has been widely used and has already made a huge impact on the study of many important biological systems and uncovered thousands of differentially expressed proteins (DEPs) closely related to different biological processes [11], [12], [13], [14]. There is no doubt that further identification of the putative DEP’s phosphorylation modification would increase our understanding of the regulatory mechanism(s) present in various biological processes. Currently, one easy way to link the two set of data between large scale phosphoproteomics studies and the 2-DE-based comparative proteomics analyses is searching the protein accession numbers of the two dataset and assign the phosphorylation information to the DEPs having the same accession numbers, whereas some important information could be confused in this process because the same phosphopeptide may be mapped to two or more DEP isoforms [15]. In contrast, directly analyzing the phosphorylation modification of specific DEPs would avoid this problem, though the procedure may be difficult because the signal of phosphopeptide is often concealed in bulks of non-phosphopeptides [16], [17]. If the phosphopeptides from known DEPs could be firstly located in the mass spectra, putative phosphosites could therefore be identified by mass spectrometer from the spots displayed on 2-DE gels without restarting a new gel-free phosphoproteomics experiment. As a consequence, the identified phosphorylation sites from related important DEPs may provide enough information to guide further functional studies.

The economically important fibers of upland cotton (*Gossypium hirsutum* L.*)* are single-celled trichomes initiated from individual epidermal cells of the ovule on the day of anthesis. After undergoing a period of fast elongation (approximately 15 days) and a secondary wall deposition period (approximately 20 days), the single-celled trichomes finally mature into spinnable fibers [12]. In addition to their economic importance, cotton fibers provide an excellent model for studying the mechanism of cell growth and elongation in plants. Our previous comparative proteomics studies have revealed that the abundances of 235 important identified proteins display significantly dynamic changes during the cotton fiber elongation process, suggesting that these important proteins are closely related to fiber elongation [12], [18]. Knowledge of phosphorylation events and their regulation is crucial to understanding the functional biology of cell growth and elongation. Therefore, characterization of reversible protein phosphorylation of these important DEPs in elongating cotton fiber cells is of great importance.

In the present study, we successfully identified a total of 40 unique phosphorylation sites of 40 phosphoproteins from the 235 known DEPs found in elongating cotton fiber cells [12], [18], including 25 new phosphorylation sites in plant cells, using a phosphosite identification approach incorporating FindMod prediction combined with MALDI TOF/TOF analysis. The results also indicate that some different isoforms of the identical DEP revealed in the 2-DE-based proteomics analysis could be annotated by phosphorylation events. In summary, our results provide the plant proteomics field an example for direct identification of phosphorylation sites from known protein spots on 2-D gels and also a valuable resource for the study of phosphorylation proteins and molecular regulation mechanisms during cotton fiber elongation.

## Materials and Methods

### Plant Materials

Upland cotton (*Gossypium hirsutum* L. cultivar CRI 35) growth was the same as previously described [12]. Fiber collected at selected stages (5, 10, 15, 20, 25 DPA) was frozen and stored in liquid nitrogen immediately after harvest until extraction of protein and RNA.

### Protein Extraction and 2-DE

Protein extraction was performed as previously described [12]. For 2-DE, one milligram of protein was loaded onto a nonlinear IPG Drystrip (pH 3–10, 24 cm, GE Healthcare, Piscataway NJ, USA) and then focusing was carried out for a total of 75 kVh. The strips were subsequently placed on the top of vertical 12.5% SDS-polyacrylamide self-cast gels and subjected to electrophoresis using an Ettan™ DALT system (GE Healthcare). The resulting gels were visualized by staining with Colloidal Coomassie Blue G-250.

### Western Blotting Assay of Protein Phosphorylation

For western blotting assays of total protein phosphorylation, 20 µg of proteins for each sample were denatured in 6× SDS-PAGE sampling buffer [1×: 0.0625 M Tris-HCl pH 6.8, 10% (v/v) glycerol, 5% (v/v) 2-mercaptoethanol, 2% SDS, and 0.001% (w/v) bromophenol blue] by boiling in a water bath for 5 min, then separated by 12% SDS-PAGE and transferred onto a PVDF membrane. The first and secondary antibodies used were phosphoserine/threonine/tyrosine antibody (Abcam ab15556; Abcam, Hong Kong, China) and goat anti-rabbit antibody conjugated to horseradish peroxidase (Catalogue No.0102; GeneSci, Beijing, China). The signals were revealed with the Lumi-light western blotting substrate (Catalogue No.12015200001; Roche, Penzberg, Germany). For western blotting assays of protein phosphorylation after 2-DE separation, only gel sections (5×7 cm) containing the largest number of proteins were used.

### FindMod Analysis of Possible Phosphopeptides

The FindMod analysis of the phosphopeptides followed the protocol provided at the website (http://web.expasy.org/findmod/) [19]. Before submission for FindMod analysis, the raw MS data of each DEP (http://world-2dpage.expasy.org/repository/0046/) was submitted to the Peakeraser software (http://www.gpmaw.com/html/peakerazor.html) to filter any trypsin and keratin peaks. Then, the amino acid sequence and filtered peaklist of the corresponding DEPs were run in FindMod. The operation parameters were set as follows: check unmatching peptides for potential post-translational modifications (max 1 within one peptide), all peptide masses are [M+H]^+^ and monoisotopic with cysteines treated with iodoacetamide, mass tolerance ±0.1 Da, trypsin as digestion enzyme, and allow for 1 missed cleavage site.

### MS/MS Identification of Phosphopeptides

MS/MS identification of phosphopeptides was conducted with a 4800 MALDI TOF/TOF™ Analyzer (Applied Biosystems, Framingham, USA). Tryptic digests of the different DEPs were redissolved in 0.1% TFA/50% acetonitrile and mixed 1∶1 with a matrix consisting of 20 mg/mL DHCA, 50% acetonitrile, and 5 mM NH_4_HPO_4_ prior to spotting on a sample plate. The sample plate was externally calibrated using the Mass Standards Kit (Applied Biosystems, Framingham, CA). The possible phosphopeptide MALDI-TOF/TOF spectra were acquired manually through inputting the peptide mass in the precursor mass window. MS/MS spectra were accumulated with at least 2000 laser shots. The obtained MS/MS spectra were searched against a *Gossypium* peptide sequence database annotated from a *Gossypium* EST database (downloaded from NCBI, http://www.ncbi.nlm.nih.gov, release date December 9, 2010, including 507,959 EST sequences and 62,267,048 residues) using MASCOT 2.2 (Matrix Science, Oxford, UK) with the following settings: enzyme, trypsin; MS tolerance, 0.3 Da; MS/MS tolerance, 0.5 Da; maximum number of missed cleavages, 1; peptide charge of 1+; fixed modifications were carbamidomethylation of Cys; variable modifications were oxidation of Met, phosphorylation and sulfation of Ser, Thr and Tyr. Peptides with an >15 ion score were determined to have significant homology (p<0.1) instead of a random event. Stringent manual analyses of spectra were then performed by hand to further validate the identification confidence of these peptdies. Furthermore, two technical repeats of the MS/MS were performed to confirm the identified phosphopeptides. The MASCOT search raw data of successfully identified phosphopeptides were deposited in PRIDE database with an accession number 27945 (http://tinyurl.com/cxs5p5z, Username: review58953, Password: qjGyQJFF) [20].

### Bioinformatics Analysis of Phosphoproteins and Phosphopeptides

The phosphoprotein function classification was the same as described in our proteomics analysis of elongating cotton fibers [18]. Consensus sequence analysis of the 48 phosphopeptides surrounding the three phosphorylation amino acid sites (serine, threonine, and tyrosine) were created and displayed using the Weblogo server (http://weblogo.berkeley.edu/). The determination of the novelty of the identified phosphorylation sites was performed through phosphopeptide BLAST searching of the P^3^DB database (http://p3db.org/) [21]. Any phosphorylation site presenting a 0 BLAST match at an E-value threshold of 100 was regarded as a new identified phosphorylation site.

## Results and Discussion

### Phosphorylation Status and Phosphosite Screening Strategy of Cotton Fiber Proteins Separated on 2-D Gels

In our previous comparative proteomics studies of elongating cotton fibers, 235 proteins were identified to be differentially expressed during the elongation process ranging from 5 to 25 days post anthesis (DPA), providing strong evidence that these important proteins are closely related to fiber elongation [14], [18]. Additionally, the 235 previously identified proteins represent 179 unique proteins (*Unigene*), suggesting that a considerable number of isoforms exist, and some isoforms were presumed to be phosphorylated or dephosphorylated on the basis of isoelectric point (pI) and molecular weight (Mw) drift on 2-DE gels [12], [18]. In order to confirm the possible phosphorylation status of these DEPs, cotton fiber total proteins, which were extracted according to the same method described previously [14] at five time points (5, 10, 15, 20, 25 DPA) of the elongation process, were subjected to SDS-PAGE and immunoblotted with phosphoserine/threonine/tyrosine antibody. As shown in Figure S1-A, many phosphoproteins could be recognized by the antibody in all 5 samples, suggesting that the phosphorylation modification is widespread in cotton fiber cells. Besides, signal intensity of a 30 kDa phosphoprotein recognized by the antibody were stronger at 5 and 10 DPA than 15–25 DPA, whereas the signal intensity of phosphoproteins around 40 kDa were stronger at 15, 20 and 25 DPA than 5–10 DPA (Figure S1-A), unambiguously implying that the reverse phosphorylation of proteins is dynamic during the cotton fiber elongation process. Similar results were obtained in 2-D western blot results. In the 7 cm×5 cm gel slice area, five DEP spots (spot No.65, No.119, No.135, No.138, and No.200) were recognized by the phosphoserine/threonine/tyrosine antibody and displayed different signal intensity (Figure S1-B), suggesting that the phosphorylation modification extent of distinct DEP spots are different. These results also implied that phosphorylation modification are well conserved in protein extraction and 2-DE process, suggesting that directly identification of phosphorylation sites from DEPs separated on 2-D gels is technically possible.

**Figure 1:**
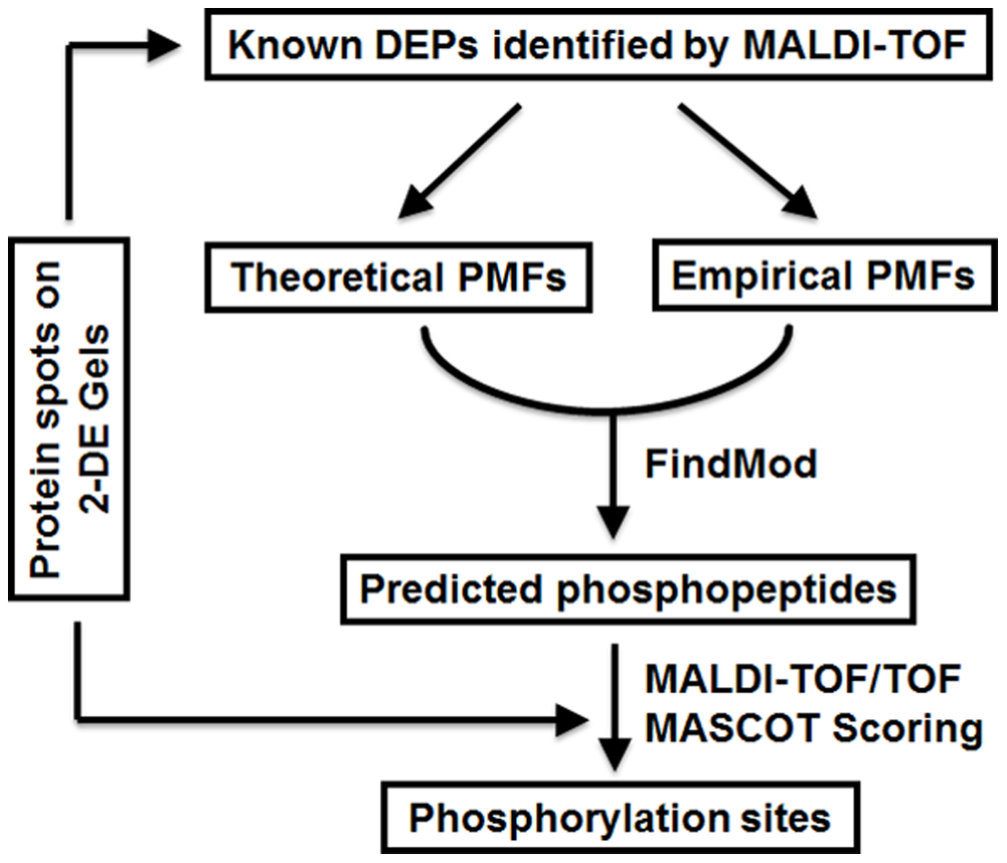
Flowchart of the 2-DE-based phosphorylation site identification method. Mass spectra and accurate amino acid sequence of the selected protein are analyzed by FindMod tool to predict the possible phosphopeptides, which are further confirmed by MS/MS and MASCOT scoring to elucidate the precise phosphorylation sites.

Although many technical innovations were applied in large-scale phosphoproteomics analyses, the ability to comprehensively identify phosphorylation sites of a selected protein remains a hurdle in 2-DE-based proteomics approach, especially from limited amounts of protein samples. Theoretically, phosphorylation sites can be first screened by prediction software tools and then identified using mass spectrometer according to the predicted phosphopeptide data of any given protein. A series of software tools, including MOWSE [22], [23], FindPept [24] and FindMod [19] have been designed to facilitate the screening of covalent modifications such as phosphorylation from raw PMF data from selected proteins and were proven to be able to guide the identification of physiologically relevant phosphorylation sites from limited starting material. For example, a combined MS and bioinformatics approach was successfully applied to map phosphorylation sites of endogenous proteins in rat [25]. Similarly, we attempt here to employ a combined FindMod software prediction and MALDI TOF/TOF analysis strategy to identify the phosphorylation sites of 235 DEPs previously identified from elongating cotton fiber cells. The prediction/identification-combined workflow is outlined in Figure 1. Briefly, the software tool FindMod was used to predict the putative phosphopeptides of any selected protein by examining the peakeraser-filtered PMF data for mass differences between the empirical and theoretical peptides, and then the manual filtered predicted phosphopeptides were individually analyzed in MS/MS mode of MALDI TOF/TOF mass spectrometer using trypsin-digested DEP samples from the re-prepared 2-D gels. The obtained MS/MS spectra were further searched against database using MASCOT to evaluate their confidence. In this way, old data of the former 2-DE-based proteomics study could be reused to directly identify phosphorylation sites from known DEPs on 2-D gels without restarting a new gel-free phosphoproteomics analyses.

**Figure 2:**
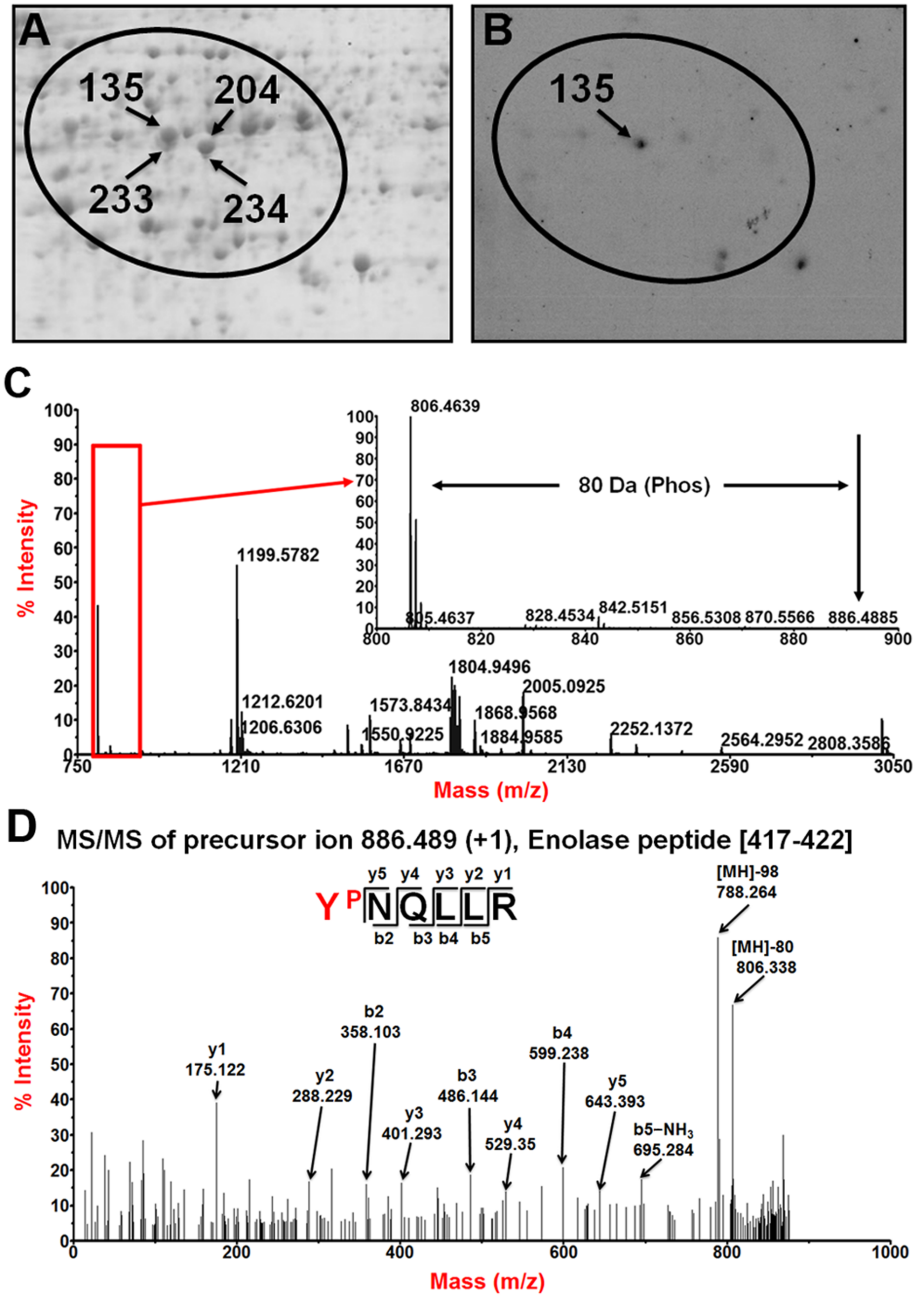
Isoforms of cotton fiber enolase protein are phosphorylated at different levels. A) A 2-DE map of total proteins in 10 DPA cotton fibers. The four isoforms of cotton fiber enolase protein are shown in the 7 cm×5 cm gel slice of the 24 cm×20 cm raw 2-D gel. B) Western blot analysis of cotton fiber protein phosphorylation after 2-DE separation. One isoform of cotton fiber enolase protein (spot 135) recognized by the antibody is shown. C) PMF mass spectra of trypsin digest of the spot 135 isoform of enolase protein displaying the non-phosphopeptide (Mw 806.4639) and the predicted phosphopeptide (Mw 886.4885). D) MS/MS spectra of the spot 135 isoform of the enolase protein phosphopeptide Y^P^NQLLR identified by MALDI TOF/TOF and MASCOT scoring.

The practicality to identify phosphorylation sites from DEPs using raw MS data-based phosphopeptide prediction combined with further MS/MS analysis was firstly assessed. As shown in Figure 2-A, four DEP spots in our previous comparative proteomics study of elongating cotton fiber cells were identified as different isoforms of the same enolase protein (ENO) [12], [18]. Following the workflow procedure in Figure 1, the peakeraser-filtered PMF data of the four isoforms were scanned with FindMod to generate a short list of putative phosphopeptides (Table S1) that can be subsequently verified by MS/MS analysis. Then the manual filtered FindMod-predicted 10 possible phosphopeptides were all analyzed in MS/MS mode of MALDI TOF/TOF mass spectrometer. The obtained 10 MS/MS spectra were searched against a *Gossypium* peptide database using MASCOT. The results indicated that the phosphopeptide AAVPSGASTGIY^P^EALELR was predicted and identified in three of the four isoforms (spot No.135, No. 204, and No. 233) (Table 1), but only the spot No.135 isoform had another phosphopeptide Y^P^NQLLR predicted (Figure 2-C) and identified (Figure 2-D and Table 1), which was in partial agreement with the 2-D western blot result that only spot No.135 isoform could be recognized by the phosphoserine/threonine/tyrosine antibody (Figure 2-B). The possible reasons why the AAVPSGASTGIY^P^EALELR phosphopeptide was unable to be recognized by the phosphoserine/threonine/tyrosine antibody are that the stoichiometry of this phosphosite is too low to be detected or the mouse-derived phospho-antibody used here is not specific for this site. Anyway, these results clearly illustrated that prediction/identification-combined method is feasible to successfully identify the phosphorylation sites of cotton fiber proteins displayed on 2-D gels.

**Table 1:** Information of the protein phosphorylation sites identified in this study.

Spot No.	Protein Name	NCBI accession No.	Phosphopeptide	Mascotion Score	Position in protein
**Cytoskeleton**					
8	Beta-tubulin 19	AF484959	FPGQLNS^P^DLRK*	19.32	242–252
9	Beta-tubulin 1	AF484959	FPGQLNS^P^DLRK*	30.87	242–252
69	Beta-tubulin 19	AF484959	FPGQLNS^P^DLRK*	23.61	242–252
93	annexin 1	ES808831	VPAHVPAPS^P^EDAEQLR	38.7	6–21
110	Actin-depolymerizing factor	DW517437	LGEPSQS^P^YDDFTASLPADEC^Cam^R	31.79	46–66
			IFFIAWSPDTS^P^R	70.66	85–96
**Redox Homeostasis**					
19	NADP-dependent oxidoreductase P2	ES825728	NLY^P^LSC^Cam^DPYMR	21.29	48–58
13	Catalase isozyme 1	X52135	HAEMFPIPPAVC^Cam^T^P^GR*	41.03	402–416
154	Glutathione S-transferase	ES825910	KHVSAWWDDIS^P^SRPSWQK*	27.79	189–206
156	GSH-dependent dehydroascorbate reductase1	ES798668	KWTVPESLT^P^NVR	27.68	169–180
**Structural and Storage Componets**					
162	MLP-like protein 31	CD485625	EVVEAVDPDKNLVT^P^FR*	52.95	74–89
**One-carbon Metabolism**					
48	5-methyltetrahydropteroyltriglutamate–homocysteine methyltransferase	ES832618	WAVHS^P^FR	15.9	616–622
49	5-methyltetrahydropteroyltriglutamate–homocysteine methyltransferase	ES832618	WAVHS^P^FR	17.18	616–622
			YGAGIGPGVYDIHS^P^PR*	91.31	679–694
77	S-adenosylmethionine synthetase 1	EF643509	FVIGGPHGDAGLT^P^GR	78.06	238–252
**Energy Metabolism**					
66	Dihydrolipoyl dehydrogenase	ES815537	LGSEVTVVEFAPDIVPS^P^MDAEIR	116.46	238–260
117	Enolase	AY297757	AAVPSGASTGIY^P^EALELR*	38.48	36–53
135	Enolase	AY297757	AAVPSGASTGIY^P^EALELR*	32.47	36–53
			Y^P^NQLLR	21.29	417–422
204	Enolase	AY297757	AAVPSGASTGIY^P^EALELR*	67.95	36–53
233	Enolase	AY297757	AAVPSGASTGIY^P^EALELR*	27.03	36–53
**Signal Transduction**					
97	14-3-3 like protein	CO123672	DS^P^TLIMQLLR	36.9	222–231
**Transport**					
60	Putative importin alpha protein	ES809347	GKPPT^P^PFEQVKPALPVLR*	112.55	235–252
194	Putative importin alpha protein	ES809347	GKPPT^P^PFEQVKPALPVLR*	71.48	235–252
65	Plasma ATP synthase subunit beta	ES810918	T^P^DHFLPIHR	20.58	186–194
201	Plasma ATP synthase subunit beta	ES810918	NLQDIIAILGMDELS^P^EDDKLTVAR*	29.74	461–484
200	Rab GDP dissociation inhibitor	ES843277	LYAES^P^LAR	27.2	210–217
			YLDEPALDT^P^VKR*	28.21	196–207
**Protein Folding and Assembly**					
160	Peptidyl-prolyl cis-trans isomerase	ES800916	VFFDMT^P^IGGQPAGR	57.52	7–20
112	Peptidyl-prolyl cis-trans isomerase	ES800916	VFFDMT^P^IGGQPAGR	57.64	7–20
			IVMELFADC^Ca^mT^P^PR	24.34	21–32
			VIPNFMC^Cam^QGGDFT^P^AGNGTGGESIYGSK*	44.69	64–90
118	26S proteasome ATPase subunit RPT5a	CO081538	T^P^MLELLNQLDGFSSDER	30.55	293–309
180	Heat shock protein 70	ES808708	SKFES^P^LVNHLIER	39.77	347–359
187	T-complex protein 1, theta subunit	ES797476	LSQPKPDDLGFVDS^P^ISVEEIGGSR	31.02	337–360
189	Chaperonin CPN60, mitochondrial	ES827612	MISTSEEIAQVGTIS^P^ANGER	69.24	168–187
**Non-Energy Carbohydrate Metabolism**					
98	UDP-L-rhamnosesynthase	EE592948	LC^Cam^ES^P^QGIDYEYGSGR*	29.65	31–45
			TNVVGT^P^LTLADVC^Cam^R	34.36	89–102
125	Putative transketolase	ES817011	ALPTYTPES^P^PADATR*	26.23	424–438
**Lipid and Secondary Metabolism**					
30	NADPH-dependent mannose 6-phosphate reductase	ES808353	SIGIS^P^NYDIFLTR	122.49	156–168
55	Betaine-aldehyde dehydrogenase	AY461804	GKDWAT^P^APGAVR	47.36	63–74
89	Phenylcoumaran benzylic ether reductase-like protein	ABN12322	FFPS^P^EFGMDVDKNNAVEPAK*	89.16	108–127
**Amino Acid Metabolism**					
84	Phosphoserine aminotransferase	ES820382	NVGPS^P^GVC^Cam^IVIVR	20.44	256–268
134	Ketol-acid reductoisomerase	DW226411	GVSFMVDNC^Cam^STT^P^AR	20.46	500–513
212	Glutamine synthetase	EU223825	IIAEYIWIGGS^P^GMDLR	51.58	19–34
137	Serine hydroxymethyltransferase	ACJ11726	ISAVSIFFETMPY^P^R	28.57	186–199
**Unknown**					
1	TIM-barrel enzyme family protein	CO129426	IHIHNAQVS^P^LMR	51.68	24–35
			QLES^P^IGFSGVQNFPTVGLFDGNFR	35.94	285–308

Abbreviations: P, phosphorylation; Cam, carboxyamidomethylation; *, phosphorylation sites already recorded in the P^3^DB database.

### Phosphopeptide Prediction from the PMF Data of 235 known DEPs in Elongating Cotton Fiber Cells

**Figure 3:**
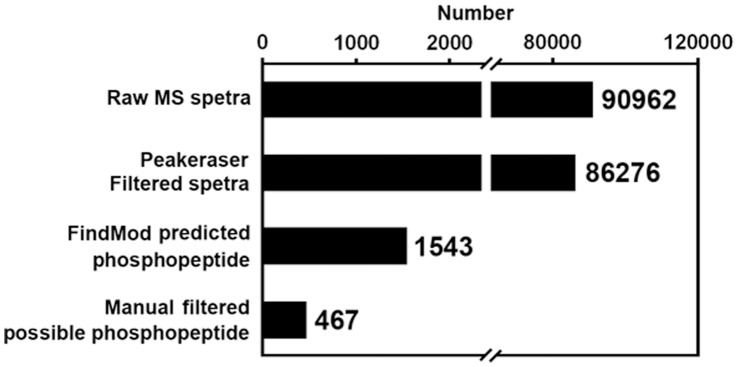
Phosphorylation site prediction of 235 differentially expressed proteins in elongating cotton fiber cells. The Peakeraser filter could partly eliminate the contamination peaks from the raw MS spectra, and further manual filter of the mistaken assignments of contaminated peptides to possible phosphopeptide largely diminished the prediction false-positive rate.

After assessing the feasibility of the phosphorylation site identification approach in practice, we then applied the approach to identify the possible phosphorylation sites from all DEPs revealed in our comparative proteomics research of elongating cotton fibers [12], [18]. For the first step, raw PMF data of 235 DEPs were all scanned with the FindMod tool, individually, to generate a list of putative phosphopeptides of selected DEPs (Table S1 and Figure 2-C). The unwanted trypsin and keratin peaks were first removed using the Peakeraser software, which eliminated 4686 contaminated spectra from the total 90962 raw MS spectra of the 235 DEPs (Figure 3 and Table S1). From the Peakeraser-filtered 86276 MS spectra of 235 DEPs, total 1543 spectra were predicted as possible phosphopeptides by FindMod (Figure 3 and Table S1).

Because some protein spots on 2-DE gels are not composed of single proteins but rather a mixture of multiple proteins [26], mistaken assignments of peptides from co-migrated low abundance proteins to phosphopeptides of target proteins are possible when the Mw of the two are approximate. How to efficiently eliminate these false predictions? In MALDI-TOF mass spectra, phosphopeptide ions and the corresponding non-phosphopeptide ions always coexist and the former are always much weaker than the latter because the phosphopeptides are sub-stoichiometric and not prone to ionization [27]. Based on this principle, the predicted phosphopeptides which do not coexist with their corresponding non-phosphopeptides or which are more intense than the corresponding non-phosphopeptides, are considered as false predictions. When the *in silico* run was ready, we thus further manually filtered the mistaken prediction through comparing the intensity of the predicted phosphopeptide ions and the corresponding non-phosphopeptide ions. This manual filter step narrowed down the number of FindMod-predicted phosphopeptides from 1543 to 467 (Figure 3 and Table S1), which largely reduced the workload of the next MS/MS identification step.

### MALDI MS/MS Identification of Phosphorylation Sites from Known DEPs in Elongating Cotton Fiber Cells

**Figure 4:**
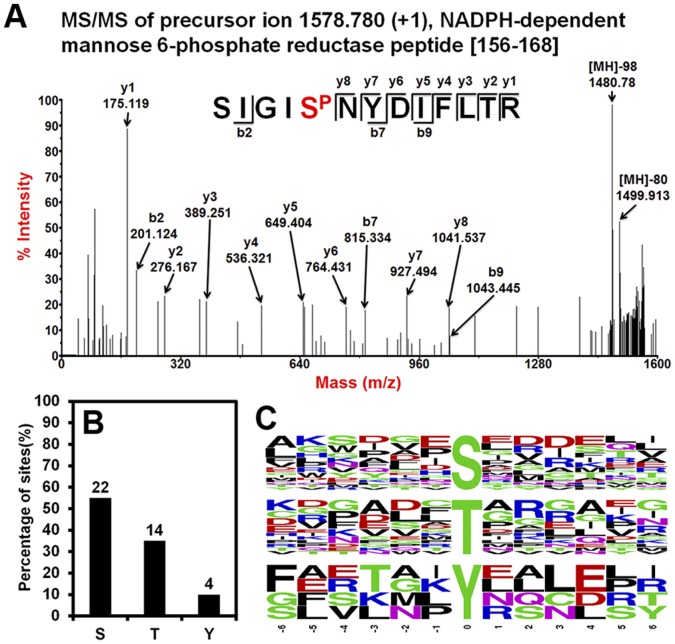
Analysis of the identified phosphorylation sites of differentially expressed proteins in elongating cotton fiber cells. A) An MS/MS spectra of one representative phosphopeptide SIGIS^P^NYDIFLTR identified using our method. B) Percentage of phosphorylated amino acids among a total of 40 unambiguously identified phosphorylation sites. C) Frequency distribution of amino acid residues surrounding phosphorylation sites at positions −6 to +6.

At the second step, the 235 DEP spots were manually excised from the new prepared 2-D gels, digested with trypsin, analyzed in MS/MS mode with a 4800 MALDI TOF/TOF™ Analyzer (Applied Biosystems, Framingham, USA) and then searched against a *Gossypium* peptide sequence database using MASCOT to discriminate the true phosphopeptides from the predictions. Among the manual filtered 467 phosphopeptides which were predicted by FindMod from the PMF data of the 235 DEPs in elongating cotton fiber cells, 48 phosphopeptides of 40 DEPs were MS/MS identified to be true phosphopeptides, all of which has an >15 MASCOT ion score (Table 1). One successfully identified phosphopeptide (peptide SIGIS^P^NYDIFLTR of cotton NADPH-dependent mannose-6-phosphate reductase protein) is shown in Figure 4-A as a representation of the identification results. Because 13 of the 48 identified phosphopeptides are actually repeatedly detected peptides for 5 phosphorylation sites (Table 1), there are total 40 unique phosphorylation sites identified.

A great amount of information was obtained from the phosphorylation site identification results. Of the 40 identified unique phosphorylation sites, 15 were found to have been identified in other plant cells via BLAST searches of the 40 phosphopeptides in the P^3^DB database [21], which means that the remaining 25 represent the first reports of new phosphorylation sites in plant cells (Table 1). In addition, 22 of the 40 unique phosphorylation sites were found to be phosphorylated on serines, whereas 14 and 4 were phosphorylated on threonines and tyrosines, respectively (Figure 4-B). Motif analysis of the 40 unique phosphorylation sites indicated that among the amino acid residues from the −6 to +6 positions of the phosphorylation sites, acidic amino acids (Glu, E and Asp, D) are enriched from the −1 to +4 positions of serine phosphorylation sites, whereas basic amino acid (Arg, R) is enriched at the +2 position of threonine phosphorylation sites (Figure 4-C), suggesting the kinase recognition motif of these two kinds of phosphorylation sites are different in cotton fiber cells [28].

### Characterization of *in vivo* Protein Phosphorylation in Elongating Cotton Fiber Cells

**Figure 5:**
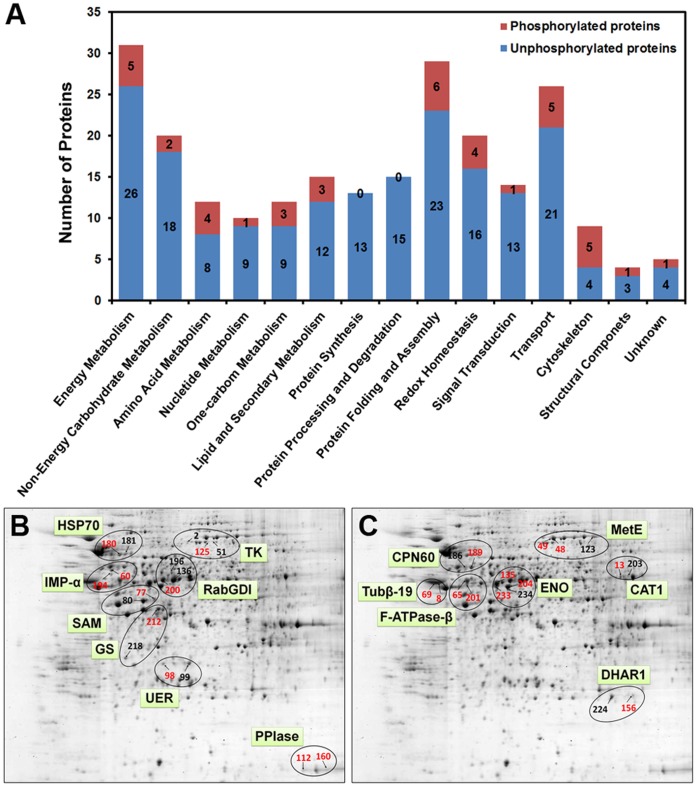
Analysis of the identified phosphorylated differentially expressed proteins in elongating cotton fiber cells. A) Functional classification and distribution of the 40 identified phosphorylated differentially expressed proteins. The number represents the number of protein spots in each functional catalog of the 40 phosphorylated DEPs and the 195 DEPs not identified as phosphorylated. B) and C) Close-up analysis of the relationship between phosphorylation modification and the possible isoforms detected by 2-DE. 35 DEP spots matched to 15 unique proteins are shown. The identified phosphoproteins are shown in red. Abbreviations: HSP70, Heat shock protein 70, chloroplast; IMP-α: Putative importin alpha protein; SAM: S-adenosylmethionine synthetase 1; GS: Glutamine synthetase; UER: UDP-L-rhamnose synthase; RabGDI: Rab GDP dissociation inhibitor; TK: Transketolase; PPIase: Peptidyl-prolyl cis-trans isomerase; CPN60: Chaperonin CPN60, mitochondrial; Tubβ-19: Beta-tubulin 19; F-ATPase-β: Plasma ATP synthase subunit beta; ENO: Enolase; CAT1: Catalase 1; DHAR1: GSH-dependent dehydroascorbate reductase; MetE: 5-methyltetrahydropteroyltriglutamate–homocysteine methyltransferase.

The pivotal significance of identifying the phosphorylation sites of the DEPs in elongating cotton fiber cells is to help us better understand the delicate mechanism of cell growth and elongation. The 40 identified phosphoproteins fall into 12 functional classes (Figure 5-A), supporting the notion that phosphorylation regulates diverse cellular processes. Among the 12 classes, protein folding and assembly, cytoskeleton and transport classes contained the higher ratio of phosphorprotein, suggesting these proteins might need more delicate regulation during the fiber elongation process. The 17.5% (40/235) ratio of phosphoproteins in our dataset is lower than the predicted 30% ratio in eukaryotes [29] but is acceptable because the ratio of phosphoprotein/total protein of large-scale plant phosphoproteomics analyses are also much lower than 30% [6], [30].

In our previous research, we reported that many biochemical pathways were significantly regulated during the cotton fiber elongation process [18], but the detailed molecular mechanisms of these regulations remained unknown. Based on our phosphopeptide identification results, we could now further extend our understanding of these processes to a molecular regulation level. Enolase (ENO), enzyme involved in glycolysis, which catalyzes the transformation of 2-phosphoglycerate to phosphoenolpyruvate, was identified as phosphorylated (Table 1). This finding is in full agreement with known reports that ENO is a phosphoprotein [31], indicating that glycolysis is regulated at this step through phosphorylation during cotton fiber elongation process. Transketolase (TK), a key enzyme linking the glycolysis and pentose phosphate pathways [32] was identified as a phosphoprotein. In addition, UDP-L-rhamnose synthase (UER), which participate in the synthesis of pectin [33], was also identified as phosphorylated. These results indicate that carbohydrate metabolism pathways, which play diverse and important roles in the cotton fiber elongation process [34], may be regulated by reverse phosphorylation of important enzymes.

Combined with the superior separation ability of 2-DE and the accuracy of MS identification, the different isoforms of same proteins can be mapped on a 2-DE gel [12], [35]. Identification of the post-translation modification differences among these isoforms could then explain how they formed. This information usually cannot be easily obtained using the shotgun proteomics method but is a straightforward result of our phosphorylation site identification method. As reported previously, there are a total of 40 proteins having 2 or more isoforms detected in the 2-DE gels of elongating cotton fiber total protein [12], [18], of which 15 were found to have phosphorylated isoforms in this study (Figures 5-B and C). Among the 15 proteins, 11 have only two isoforms, and 7 of those, including UDP-L-rhamnose synthase (UER), have one isoform phosphorylated and another unphosphorylated isoform. The remaining 4, including beta-tubulin 19 (Tubβ19), have two isoforms that are both phosphorylated. Among the 4 proteins having 3 or more isoforms, only one phosphorylated isoform was found for transketolase (TK) and Rab GDP dissociation inhibitor (RabGDI). Two phosphorylated isoforms were found for 5-methyltetrahydropteroyltriglutamate–homocysteine methyltransferase (MetE), and three phosphorylated isoforms were found for enolase (ENO) (Table 1). These results further support the finding that reverse phosphorylation of proteins is widespread and dynamic during the cotton fiber elongation process (Figure S1 and Table 1).

### Conclusion

Cotton fiber elongation is a dynamic process accompanied by major protein changes at different elongation stages. How these proteins are delicately regulated to drive the fiber elongation remains to be elucidated. In this study, we reported, for the first time, on cotton fiber phosphoproteome research that focuses on the 235 proteins differentially expressed during the elongation process. In total, 40 unique phosphorylation sites were successfully identified from the 235 known DEPs previously revealed in the 2-D gels of elongating cotton fiber cells. Phosphorylation of many important proteins, such as enolase and UDP-L-rhamnose synthase, which were already known to be differentially expressed and play important roles in cotton fiber elongation process, was identified for the first time. In summary, our study presents a valuable resource for future functional studies of phosphorylated proteins in cotton fiber elongation related research.

## Supporting Information

Figure S1 **Western blot analysis of cotton fiber total protein phosphorylation after Tris-phenol extraction followed with 1-D and 2-D-SDS-PAGE.** A. Cotton fiber total proteins sampled from 5 stages of the elongation process were run on an SDS-PAGE (left panel) and analyzed by western blot using an anti-phosphorylation antibody (right panel). B. Cotton fiber total proteins sampled from 5 stages of the elongation process were combined and run on an 2-D gel (left panel), a 7 cm×5 cm gel slice were cut from the gel and analyzed by western blot using an anti-phosphorylation antibody (right panel).
(DOCX)

Table S1 Statistical information on the phosphorylation site prediction process.
(DOCX)
