# Supplementary material for: Mass Spectrometric Identification of In Vivo Phosphorylation Sites of Differentially Expressed Proteins in Elongating Cotton Fiber Cells
Source: PLoS One. 2013 Mar 13;8(3):e58758. doi: 10.1371/journal.pone.0058758 (PMC3596310; doi:10.1371/journal.pone.0058758)
Supplement: Figure S1 — Western blot analysis of cotton fiber total protein phosphorylation after Tris-phenol extraction followed with 1-D and 2-D-SDS-PAGE. A. Cotton fiber total proteins sampled from 5 stages of the elongation process were run on an SDS-PAGE (left panel) and analyzed by western blot using an anti-phosphorylation antibody (right panel). B. Cotton fiber total proteins sampled from 5 stages of the elongation process were combined and run on an 2-D gel (left panel), a 7 cm×5 cm gel slice were cut from the gel and analyzed by western blot using an anti-phosphorylation antibody (right panel). (DOCX) [file pone.0058758.s001.docx]

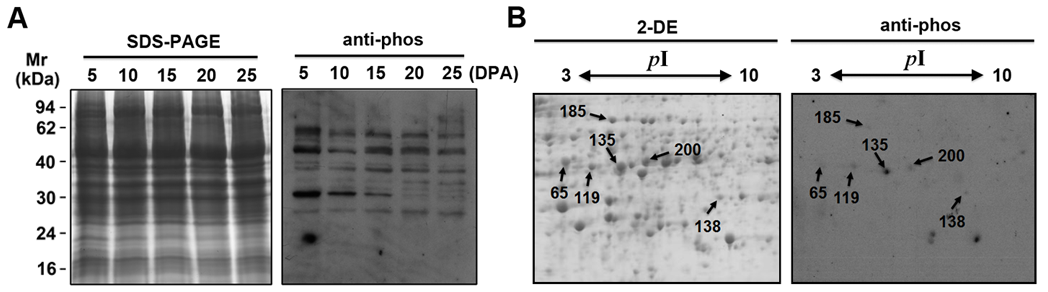


**Figure S1. Western blot analysis of cotton fiber total protein phosphorylation after Tris-phenol extraction followed with 1-D and 2-D-SDS-PAGE.**

A. Cotton fiber total proteins sampled from 5 stages of the elongation process were run on an SDS-PAGE (left panel) and analyzed by western blot using an anti-phosphorylation antibody (right panel). B. Cotton fiber total proteins sampled from 5 stages of the elongation process were combined and run on an 2-D gel (left panel), a 7 cm × 5 cm gel slice were cut from the gel and analyzed by western blot using an anti-phosphorylation antibody (right panel).
