# Supplementary material for: Mass Spectrometric Identification of In Vivo Phosphorylation Sites of Differentially Expressed Proteins in Elongating Cotton Fiber Cells
Source: PLoS One. 2013 Mar 13;8(3):e58758. doi: 10.1371/journal.pone.0058758 (PMC3596310; doi:10.1371/journal.pone.0058758)
Supplement: Table S1 — Statistical information on the phosphorylation site prediction process. (DOCX) [file pone.0058758.s002.docx]

**Table S1. Statistical information on the phosphorylation site prediction process**

| **Spot No.** | **Number of raw MS spectra** | **Number of *Peakeraser* filtered spectra** | **Number of *FindMod* predicted phosphopeptide** | **Number of manual filtered possible phosphopeptide** |
| --- | --- | --- | --- | --- |
| 1 | 609 | 571 | 10 | 3 |
| 2 | 294 | 284 | 3 | 1 |
| 3 | 596 | 574 | 10 | 4 |
| 4 | 538 | 505 | 1 | 1 |
| 5 | 358 | 331 | 5 | 1 |
| 6 | 484 | 462 | 6 | 1 |
| 7 | 386 | 372 | 6 | 1 |
| 8 | 238 | 230 | 8 | 4 |
| 9 | 428 | 408 | 12 | 6 |
| 10 | 492 | 466 | 7 | 2 |
| 11 | 236 | 227 | 5 | 2 |
| 12 | 493 | 465 | 9 | 4 |
| 13 | 349 | 337 | 12 | 4 |
| 14 | 872 | 837 | 21 | 4 |
| 15 | 150 | 141 | 3 | 1 |
| 16 | 434 | 405 | 1 | 0 |
| 17 | 483 | 429 | 6 | 1 |
| 18 | 289 | 273 | 6 | 3 |
| 19 | 546 | 519 | 5 | 2 |
| 20 | 364 | 347 | 9 | 5 |
| 21 | 184 | 175 | 2 | 1 |
| 22 | 542 | 511 | 8 | 2 |
| 23 | 306 | 296 | 6 | 1 |
| 24 | 306 | 297 | 5 | 0 |
| 25 | 194 | 186 | 1 | 0 |
| 26 | 596 | 567 | 1 | 0 |
| 27 | 135 | 126 | 2 | 1 |
| 28 | 303 | 290 | 6 | 3 |
| 29 | 301 | 286 | 2 | 1 |
| 30 | 439 | 420 | 17 | 6 |
| 31 | 429 | 404 | 4 | 0 |
| 32 | 393 | 360 | 4 | 1 |
| 33 | 165 | 149 | 2 | 2 |
| 34 | 157 | 141 | 1 | 0 |
| 35 | 361 | 326 | 4 | 2 |
| 36 | 395 | 371 | 1 | 1 |
| 37 | 327 | 308 | 2 | 1 |
| 38 | 321 | 310 | 2 | 2 |
| 39 | 391 | 379 | 16 | 6 |
| 40 | 416 | 388 | 10 | 3 |
| 41 | 269 | 256 | 2 | 1 |
| 42 | 88 | 84 | 2 | 0 |
| 43 | 233 | 220 | 2 | 0 |
| 44 | 412 | 400 | 10 | 5 |
| 45 | 361 | 355 | 8 | 1 |
| 46 | 542 | 514 | 18 | 7 |
| 47 | 441 | 420 | 10 | 5 |
| 48 | 274 | 268 | 5 | 1 |
| 49 | 858 | 819 | 28 | 6 |
| 50 | 228 | 191 | 2 | 0 |
| 51 | 824 | 791 | 7 | 1 |
| 52 | 817 | 779 | 18 | 4 |
| 53 | 384 | 369 | 4 | 0 |
| 54 | 413 | 397 | 6 | 2 |
| 55 | 523 | 508 | 6 | 1 |
| 56 | 330 | 303 | 9 | 1 |
| 57 | 458 | 425 | 20 | 5 |
| 58 | 252 | 239 | 6 | 1 |
| 59 | 445 | 424 | 4 | 0 |
| 60 | 500 | 482 | 6 | 2 |
| 61 | 279 | 267 | 5 | 3 |
| 62 | 311 | 298 | 1 | 0 |
| 63 | 465 | 445 | 5 | 1 |
| 64 | 244 | 236 | 5 | 1 |
| 65 | 194 | 187 | 8 | 4 |
| 66 | 396 | 385 | 16 | 10 |
| 67 | 313 | 301 | 7 | 2 |
| 68 | 270 | 254 | 2 | 0 |
| 69 | 240 | 235 | 3 | 3 |
| 70 | 464 | 449 | 3 | 1 |
| 71 | 354 | 339 | 5 | 1 |
| 72 | 388 | 351 | 16 | 3 |
| 73 | 233 | 223 | 5 | 1 |
| 74 | 266 | 247 | 3 | 1 |
| 75 | 162 | 153 | 1 | 1 |
| 76 | 332 | 320 | 3 | 2 |
| 77 | 568 | 538 | 10 | 1 |
| 78 | 338 | 328 | 5 | 2 |
| 79 | 303 | 291 | 3 | 0 |
| 80 | 147 | 143 | 1 | 0 |
| 81 | 641 | 616 | 16 | 3 |
| 82 | 191 | 175 | 1 | 0 |
| 83 | 492 | 476 | 6 | 1 |
| 84 | 417 | 400 | 10 | 1 |
| 85 | 281 | 275 | 4 | 1 |
| 86 | 151 | 144 | 3 | 1 |
| 87 | 507 | 486 | 5 | 3 |
| 88 | 515 | 499 | 9 | 4 |
| 89 | 415 | 389 | 6 | 2 |
| 90 | 573 | 552 | 8 | 5 |
| 91 | 223 | 215 | 2 | 0 |
| 92 | 368 | 354 | 5 | 1 |
| 93 | 214 | 205 | 6 | 3 |
| 94 | 404 | 373 | 1 | 0 |
| 95 | 260 | 245 | 3 | 1 |
| 96 | 455 | 426 | 6 | 2 |
| 97 | 147 | 141 | 5 | 2 |
| 98 | 399 | 371 | 8 | 4 |
| 99 | 302 | 272 | 4 | 1 |
| 100 | 683 | 648 | 9 | 5 |
| 101 | 259 | 243 | 3 | 1 |
| 102 | 190 | 168 | 3 | 1 |
| 103 | 179 | 173 | 1 | 0 |
| 104 | 504 | 465 | 2 | 0 |
| 105 | 308 | 284 | 5 | 2 |
| 106 | 477 | 458 | 7 | 3 |
| 107 | 530 | 497 | 3 | 1 |
| 108 | 221 | 206 | 1 | 1 |
| 109 | 205 | 193 | 1 | 1 |
| 110 | 334 | 320 | 2 | 2 |
| 111 | 304 | 282 | 3 | 1 |
| 112 | 162 | 156 | 1 | 1 |
| 113 | 375 | 354 | 6 | 3 |
| 114 | 563 | 538 | 4 | 1 |
| 115 | 140 | 126 | 1 | 0 |
| 116 | 519 | 502 | 10 | 4 |
| 117 | 428 | 413 | 7 | 5 |
| 118 | 538 | 510 | 16 | 3 |
| 119 | 129 | 125 | 2 | 2 |
| 120 | 533 | 509 | 10 | 0 |
| 121 | 276 | 260 | 3 | 2 |
| 122 | 393 | 379 | 16 | 6 |
| 123 | 266 | 257 | 6 | 1 |
| 124 | 324 | 307 | 1 | 0 |
| 125 | 562 | 539 | 14 | 3 |
| 126 | 584 | 555 | 24 | 3 |
| 127 | 548 | 522 | 19 | 7 |
| 128 | 160 | 146 | 4 | 0 |
| 129 | 316 | 300 | 16 | 4 |
| 130 | 379 | 364 | 6 | 5 |
| 131 | 303 | 293 | 6 | 2 |
| 132 | 388 | 367 | 13 | 3 |
| 133 | 502 | 478 | 12 | 2 |
| 134 | 422 | 406 | 10 | 4 |
| 135 | 420 | 405 | 8 | 3 |
| 136 | 374 | 359 | 3 | 0 |
| 137 | 541 | 512 | 9 | 2 |
| 138 | 93 | 87 | 1 | 1 |
| 139 | 471 | 455 | 9 | 2 |
| 140 | 319 | 306 | 1 | 0 |
| 141 | 284 | 271 | 2 | 1 |
| 142 | 465 | 443 | 12 | 5 |
| 143 | 498 | 476 | 9 | 2 |
| 144 | 326 | 308 | 10 | 0 |
| 145 | 424 | 406 | 7 | 2 |
| 146 | 261 | 248 | 2 | 2 |
| 147 | 419 | 389 | 15 | 0 |
| 148 | 335 | 306 | 3 | 1 |
| 149 | 124 | 111 | 1 | 0 |
| 150 | 260 | 250 | 5 | 1 |
| 151 | 259 | 247 | 1 | 0 |
| 152 | 219 | 205 | 2 | 0 |
| 153 | 185 | 173 | 2 | 0 |
| 154 | 436 | 418 | 7 | 4 |
| 155 | 368 | 359 | 4 | 0 |
| 156 | 207 | 195 | 3 | 2 |
| 157 | 244 | 226 | 1 | 0 |
| 158 | 106 | 96 | 1 | 0 |
| 159 | 480 | 435 | 6 | 0 |
| 160 | 419 | 398 | 11 | 5 |
| 161 | 291 | 276 | 1 | 1 |
| 162 | 450 | 422 | 5 | 2 |
| 163 | 612 | 590 | 1 | 0 |
| 164 | 439 | 408 | 11 | 2 |
| 165 | 603 | 577 | 2 | 0 |
| 166 | 659 | 635 | 5 | 2 |
| 167 | 640 | 600 | 12 | 3 |
| 168 | 455 | 443 | 14 | 6 |
| 169 | 387 | 375 | 13 | 3 |
| 170 | 588 | 564 | 6 | 2 |
| 171 | 400 | 367 | 7 | 2 |
| 172 | 443 | 413 | 15 | 4 |
| 173 | 211 | 190 | 10 | 2 |
| 174 | 291 | 278 | 3 | 1 |
| 175 | 445 | 416 | 1 | 0 |
| 176 | 336 | 315 | 2 | 1 |
| 177 | 403 | 381 | 8 | 1 |
| 178 | 455 | 431 | 13 | 2 |
| 179 | 502 | 469 | 2 | 0 |
| 180 | 294 | 289 | 10 | 5 |
| 181 | 423 | 393 | 10 | 0 |
| 182 | 428 | 410 | 2 | 0 |
| 183 | 302 | 290 | 15 | 4 |
| 184 | 438 | 416 | 8 | 2 |
| 185 | 125 | 117 | 1 | 0 |
| 186 | 734 | 696 | 12 | 2 |
| 187 | 564 | 544 | 9 | 3 |
| 188 | 594 | 570 | 2 | 0 |
| 189 | 355 | 340 | 13 | 4 |
| 190 | 493 | 464 | 8 | 0 |
| 191 | 320 | 308 | 6 | 0 |
| 192 | 492 | 458 | 12 | 2 |
| 193 | 166 | 156 | 3 | 1 |
| 194 | 491 | 464 | 13 | 3 |
| 195 | 777 | 745 | 6 | 1 |
| 196 | 399 | 375 | 5 | 4 |
| 197 | 577 | 549 | 3 | 0 |
| 198 | 415 | 396 | 10 | 7 |
| 199 | 399 | 374 | 9 | 2 |
| 200 | 356 | 344 | 5 | 2 |
| 201 | 361 | 348 | 13 | 6 |
| 202 | 538 | 510 | 11 | 4 |
| 203 | 295 | 241 | 11 | 3 |
| 204 | 289 | 270 | 6 | 3 |
| 205 | 275 | 268 | 3 | 1 |
| 206 | 491 | 481 | 10 | 4 |
| 207 | 430 | 412 | 12 | 3 |
| 208 | 359 | 338 | 7 | 1 |
| 209 | 344 | 321 | 12 | 2 |
| 210 | 387 | 369 | 3 | 1 |
| 211 | 431 | 398 | 7 | 1 |
| 212 | 292 | 275 | 3 | 3 |
| 213 | 455 | 418 | 6 | 3 |
| 214 | 636 | 611 | 14 | 1 |
| 215 | 555 | 520 | 8 | 1 |
| 216 | 529 | 511 | 14 | 3 |
| 217 | 567 | 537 | 12 | 5 |
| 218 | 581 | 546 | 2 | 1 |
| 219 | 650 | 632 | 13 | 5 |
| 220 | 463 | 433 | 6 | 1 |
| 221 | 657 | 636 | 4 | 3 |
| 222 | 722 | 690 | 7 | 6 |
| 223 | 460 | 425 | 4 | 3 |
| 224 | 327 | 305 | 3 | 2 |
| 225 | 261 | 246 | 2 | 1 |
| 226 | 347 | 327 | 4 | 1 |
| 227 | 505 | 475 | 2 | 0 |
| 228 | 287 | 257 | 1 | 0 |
| 229 | 274 | 243 | 4 | 1 |
| 230 | 288 | 276 | 2 | 0 |
| 231 | 313 | 284 | 5 | 3 |
| 232 | 88 | 85 | 3 | 0 |
| 233 | 287 | 277 | 6 | 2 |
| 234 | 371 | 359 | 3 | 2 |
| 235 | 380 | 356 | 16 | 5 |
| **All** | **90962** | **86276** | **1543** | **467** |
